# A SHort course Accelerated RadiatiON therapy (SHARON) dose-escalation trial in older adults head and neck non-melanoma skin cancer

**DOI:** 10.1259/bjr.20211347

**Published:** 2022-04-28

**Authors:** Milena Ferro, Francesco Deodato, Marica Ferro, Giulia Panza, Milly Buwenge, Donato Pezzulla, Savino Cilla, Mariangela Boccardi, Carmela Romano, Alessandra Arcelli, Silvia Cammelli, Alice Zamagni, Alessio Giuseppe Morganti, Gabriella Macchia

**Affiliations:** Radiation Oncology Unit, Gemelli Molise Hospital, Università Cattolica del Sacro Cuore, Campobasso, Italy; Radiation Oncology Unit, Gemelli Molise Hospital, Università Cattolica del Sacro Cuore, Campobasso, Italy; Istituto di Radiologia, Università Cattolica del Sacro Cuore, Rome, Italy; Radiation Oncology Unit, Gemelli Molise Hospital, Università Cattolica del Sacro Cuore, Campobasso, Italy; Università Cattolica del Sacro Cuore, Rome, Italy; Radiation Oncology, IRCCS Azienda Ospedaliero-Universitaria di Bologna, Bologna, Italy; Department of Experimental, Diagnostic, and Specialty Medicine - DIMES, Alma Mater Studiorum Bologna University, Bologna, Italy; Radiation Oncology Unit, Gemelli Molise Hospital, Università Cattolica del Sacro Cuore, Campobasso, Italy; Medical Physics Unit, Gemelli Molise Hospital, Università Cattolica del Sacro Cuore, Campobasso, Italy; Radiation Oncology Unit, Gemelli Molise Hospital, Università Cattolica del Sacro Cuore, Campobasso, Italy; Medical Physics Unit, Gemelli Molise Hospital, Università Cattolica del Sacro Cuore, Campobasso, Italy; Radiation Oncology, IRCCS Azienda Ospedaliero-Universitaria di Bologna, Bologna, Italy; Department of Experimental, Diagnostic, and Specialty Medicine - DIMES, Alma Mater Studiorum Bologna University, Bologna, Italy; Radiation Oncology, IRCCS Azienda Ospedaliero-Universitaria di Bologna, Bologna, Italy; Department of Experimental, Diagnostic, and Specialty Medicine - DIMES, Alma Mater Studiorum Bologna University, Bologna, Italy; Radiation Oncology, IRCCS Azienda Ospedaliero-Universitaria di Bologna, Bologna, Italy; Department of Experimental, Diagnostic, and Specialty Medicine - DIMES, Alma Mater Studiorum Bologna University, Bologna, Italy; Radiation Oncology, IRCCS Azienda Ospedaliero-Universitaria di Bologna, Bologna, Italy; Department of Experimental, Diagnostic, and Specialty Medicine - DIMES, Alma Mater Studiorum Bologna University, Bologna, Italy; Radiation Oncology Unit, Gemelli Molise Hospital, Università Cattolica del Sacro Cuore, Campobasso, Italy

## Abstract

**Objectives:**

To assess feasibility and safety of a SHort-course Accelerated RadiatiON therapy (SHARON) regimen, in the treatment of non-melanoma skin cancers (NMSC) in older patients.

**Methods:**

Old patients (age ≥ 80 years) with histological confirmed non-melanoma skin cancers were enrolled. The primary endpoint was to determine the maximum tolerated dose (MTD). Radiotherapy regimen was based on the delivery of four radiotherapy fractions (5 Gy per fraction) with a twice daily fractionation in two consecutive days. Three different level of dose were administered: 20 Gy (one cycle), 40 Gy (two cycles) and 60 Gy (three cycles).

**Results:**

Thirty patients (median age: 91 years; range: 80-96) were included in this analysis. Among fourteen patients who completed the one cycle, only one (7%) experimented acute G4 skin toxicity. Twelve patients reported an improvement or resolution of baseline symptoms (overall palliative response rate: 85.8%). Nine and seven patients underwent to two and three RT cycles, respectively: of these, no G3 toxicities were recorded. The overall response rate was 100% when three cycles were delivered. The overall six-month symptom-free survival was 78.7% and 77.8% in patients treated with one course and more courses, respectively.

**Conclusions:**

Short-course accelerated radiotherapy in older patients with non-melanoma skin cancers is well tolerated. High doses seem to be more effective in terms of response rate.

**Advances in knowledge:**

This approach could represent an option for older adults with NMSC, being both palliative (one course) or potentially curative (more courses) in the aim, accordingly to the patient’s condition.

## Introduction

Non-melanoma skin cancer (NMSC), comprising of basal cell carcinoma (BCC) and squamous cell carcinoma (SCC), is by far the most frequent cancer in white population, and numerous studies have shown that incidence rates of NMSC are increasing worldwide.^1–6^ Age-specific incidence rates continuously increased between 1970 and 2016: throughout the entire period, the highest incidence rates were observed in >80 years people.^7^ Large skin lesions are often painful, bleeding and septic, causing discomfort for both patient and caregivers.^8^ In these cases, a palliative radiotherapy (RT) is indicated, although a consensus on the standard dose and fractionation is still lacking.^9,10^ For smaller lesions, conventional radiation treatments take 6 weeks to complete. Unfortunately, completion of planned treatment protocol without interruptions is not always possible because of comorbid diseases and poor patient compliance. The compliance rate and completion rate of planned treatment are far less in elderly patients.^11^ Hypofractionation, indeed, an approach where the number of fractions is reduced while increasing dose per fraction, seems to be an effective option for local control with also a tolerable treatment-related toxicity.^12^

In our experience, various trials investigating a palliative schedule treatment for cancer sites throughout the body, reported satisfying outcomes^13–19^ even in older patients.^20,21^ In particular, repeated short RT courses in head and neck cancer resulted safe and able to prolong palliative outcomes.^21^ We postulated that these results could be translated in head and neck skin cancer patients, a setting where an urgent symptoms palliation as well as an effective treatment improving symptoms control are required.

Based on this rationale, we planned a prospective phase I trial of a repeated SHort-course Accelerated RadiatiON therapy (SHARON RT) in old adults (≥80 years) with an NMSC in head and neck (H&N) region.

## Methods and materials

### Study design and endpoints

The original trial for Head and Neck cancer was approved by the Catholic University Institutional Review Board (SHARON H&N protocol # NCT03196700; local Ethical Committee: UCSC-CB-2009/31); the adaptation in head and neck NMSC of older patients was subsequently approved by the Internal Hospital Tumour Board.

This was a single-center dose-escalation trial aimed to determine the maximum tolerated dose (MTD), designated as the dose level below that in which dose-limiting toxicity (DLT) appears in at least one-third of patients. Three dose levels were considered starting from a total dose of 20 Gy, five Gy per fraction delivered two times a day in two consecutive days (first cohort). The second cohort underwent this treatment twice, one month apart, reaching a total dose of 40 Gy. Similarly, three cycles (total dose: 60 Gy) were administered to the third cohort a month apart from each other. DLT was defined as any treatment-related non-hematologic acute adverse effect graded three or higher according to the RTOG scale.^22^

At the first dose level, a minimum of six patients were treated. After the treatment of the last patient in the cohort, three months of follow-up were necessary to accurately assess acute toxicity. In the meantime, the enrolment continued. If two of the six patients at a certain dose level presented severe acute toxicity (Grade ≥ 3), other six patients were enrolled to expand the cohort up to twelve patients.

The study would have been stopped if: 1) DLT incurred in more than two patients in a not-expanded group (six patients) or 2) four or more than four patients in the larger cohort (twelve patients) have had unacceptable toxicity. In both cases, the recommended dose was the dose level below that one tested and, if not, the trial moved on to the following dose level.

The secondary endpoints were late toxicities, symptoms (pain and bleeding) response rate, QoL scores, symptoms-free survival (SFS). Survival outcomes were calculated from the first day of RT until last follow-up visit, or loss to follow-up or death.

### Major inclusion/exclusion criteria

Inclusion criteria were: histological proved non-melanoma skin cancer (NMSC) in head and neck region, age ≥ 80 years, expected survival > three months, Eastern Cooperative Oncology Group (ECOG) performance status (PS) of ≤ three, and contraindication to surgical excision or chemoradiation due to difficulty in reconstruction and/or comorbidities. Furthermore, patient’s preferences and logistical issues were considered. On the contrary, exclusion criteria were collagenopathies or previous irradiation on the same area.

Every patient signed a written consent form authorizing the therapy and the use of their data in further analyses. A comprehensive clinical history, physical examination, complete blood count, and a head and neck computed tomography (CT) scan and/or magnetic resonance imaging (MRI) were all part of the pre-treatment evaluation. Data on pain and other symptoms, performance status (ECOG), and quality of life (QoL) were collected before and after the treatment. Visual Analog self-assessment Scale (VAS) was used to measure pain, whilst pain intensity and use of analgesics were also recorded according to the International Atomic Energy Agency (IAEA) scale (Pain and Drug scores). As reported elsewhere, QoL indices were assessed by the cancer linear analog scales (CLAS1, CLAS2, and CLAS3).^23^

### Treatment

All patients underwent planning CT-simulation (5 mm increments over the region of interest) in supine position. A thermoplastic mask was used for immobilization purpose. The gross tumour volume (GTV) was identified by clinical inspection and palpation, then the lesion was marked-up with a thin wire. A diagnostic CT and/or MRI was used to assess the disease extent and the depth of invasion. The clinical treatment volume (CTV) included the GTV plus 1 cm margin, while the planning target volume (PTV) was defined by adding another isotropic 1 cm margin to the CTV (adapted if required). A surface bolus was used in all cases, to ensure adequate dose at the skin.^24^ The bolus thickness (5 or 10 mm) was chosen depending on the depth of cancer invasion. No nodal irradiation was performed. The 3D conformal radiotherapy (3D-CRT) technique was used to facilitate and faster the planning time; however, when doses to organs at risk (OARs) were unacceptable, intensity-modulated RT (IMRT) technique plan was performed and delivered. The dose was specified according to the International Commission of Radiation Units and Measurement (ICRU) 62 for 3D-CRT and ICRU 83 for IMRT plans. Because of their capacity to customize the dose distribution in lesions that can appear with a variety of shapes, dimensions, depths of invasion, and locations, photons were selected over electrons. The low-energy electrons are not really safe in multiple non-parallel beam configurations in more shallow tumour regions due to the steep dose gradients at the end of their range. Indeed, electrons would cause undesirable hotspots in the dose distribution, particularly in the irregular facial “mask regions” (nose, inner canthus of eye, ear). Furthermore, multifield photon beams with various gantry angles are more helpful in covering the PTV correctly. QUANTEC guidelines were used for dose-volume constraints of OARs, considering the equivalent dose according to the linear-quadratic model. The maximum accepted dose for spinal cord was 12 Gy (3 Gy/fraction), equivalent to 14.4 Gy (at two Gy/fraction, assuming an α/β ratio of 3) in case of a single RT cycle in order to reach 43.2 Gy in case of three RT courses. Similarly, the maximum accepted dose for optic nerve was 14 Gy (3.5 Gy/fraction) and 54.6 Gy when three RT cycles were administered. As reported above, RT treatment consisted of four fractions twice a day, in two consecutive days. To allow normal tissue repair, at least eight hours were necessary between the two daily fractions. The equivalent doses in two Gy fractions (EQD2) for late effects (α/β ratio: 3) of the three different dose levels (20 Gy, 40 Gy and 60 Gy) were 32 Gy, 64 Gy and 96 Gy, respectively.^25^ If more than one RT course were planned, subsequent cycles started one month following the earlier one. In case of tumour volume shrinkage or other anatomical changes, the contours were adapted on a new CT-simulation. Isocenter position was online checked with portal imaging and corrections were made if more than five millimetres displacement in any direction were detected.^26^

### Toxicity and symptoms response evaluation

Three weeks after every treatment all patients underwent clinical evaluation. For the second and third cohorts, severe acute toxicities or tumour progression had to be excluded during the first follow-up. Afterward, a physical examination and a monitoring blood count were performed every two months. The Radiation Therapy Oncology Group scales and the European Organization for Research and Treatment of Cancer and Radiation Therapy Oncology Group scales (EORTC-RTOG) were used to record acute and late toxicities, respectively.^22^ During follow-up visits, information about chronic effects, such as cosmesis, were recorded; however, the short life expectancy, due to age and comorbidities, overshadows late toxicities that could occur several months after the treatment. Furthermore, data about QoL, pain and drug score and symptom relief were registered. Complete palliation of bleeding was defined when no further medication was needed, and complete pain relief when a VAS score was zero. Reduction of symptom severity or a decrease in pain and drug score were defined as partial response. The sum of complete and partial response defined the overall response. Furthermore, data on bodyweight, performance status, and QoL were assessed as improved, steady, or worse compared to baseline ones. Statistical analysis was performed with SYSTAT version 11.0 (SPSS, Chicago, IL, USA). SFS was defined as the time elapsed between the treatment and symptom recurrence/worsening or date of death or last follow-up. Kaplan and Meier method^27^ was used to computed life tables and medians and the log-rank test was performed to evaluate the statistical significance.^28^

## Results

Thirty consecutive patients were enrolled between February 2010 and June 2020. In all cohorts, there was a slight predominance of female gender, with an overall median age of 91 years (range 80–96) (Table 1). Most patients had recurrent disease and Grades 2–3 pre-treatment ECOG. As expected, the most represented histotype was squamous (76.7%), followed by basal cell carcinoma (13.3%). Concerning the lesion site, the facial “mask area” was predominant, in particular the ear and pre- and retro-auricular region (33.4%), the eye and periorbital region (16.7%), and the nose (13.3%). Median tumour dimension was 4.5 cm (range 1.5–11.0 cm). Twenty-three (76.7%) patients suffered from symptoms, mainly pain (52.2%), bleeding (34.8%) or both (13%). No lymph nodal metastases were treated in this series.

**Table 1.** Patients characteristics

	Overall n. (%)	Cohort 1 n (%)	Cohort 2 n (%)	Cohort 3 n (%)
		(20 Gy)	(40 Gy)	(60 Gy)
Patients	30 (100.0)	14 (46.7)	9 (30.0)	7 (23.3)
Gender				
Male	12 (40.0)	5 (35.7)	4 (44.4)	3 (42.9)
Female	18 (60.0)	9 (64.3)	5 (55.6)	4 (57.1)
Age, years				
Median (range)	91 (80-96)	88.5 (80–96)	91 (87-95)	88 (80-96)
ECOG PS				
0–1	8 (26.7)	4 (28.6)	2 (22.2)	2 (28.6)
2–3	22 (73.3)	10 (71.4)	7 (77.8)	5 (71.4)
Charlson Comorbidity Index age related				
Median (range)	5 (4–8)	5 (4–8)	6 (4–6)	5 (4–7)
Histotype				
Squamous Cell Carcinoma	23 (76.7)	8 (57.1)	8 (88.9)	7 (100.0)
Basal Cell Carcinoma	4 (13.3)	4 (28.6)	0	0
Others	3 (10.0)	2 (14.3)	1 (11.1)	0
Tumor Site				
Ear, pre- and retro-auricular region	10 (33.4)	4 (28.7)	4 (44.4)	2 (28.6)
Eyelid-periorbital area	5 (16.7)	3 (21.4)	2 (22.2)	0
Nose	4 (13.3)	2 (14.3)	2 (22.2)	0
Mandibular area	4 (13.3)	3 (21.4)	0	1 (14.3)
Cheek	4 (13.3)	1 (7.1)	0	3 (42.8)
Forehead-temples	3 (10.0)	1 (7.1)	1 (11.2)	1 (14.3)
Presenting Symptoms^a^				
Pain	20 (66.7)	10 (71.4)	6 (66.7)	4 (57.1)
Bleeding	11 (36.7)	6 (42.9)	3 (33.3)	2 (28.6)
T stage				
2	5 (16.7)	3 (21.4)	2 (22.2)	0
3	21 (70.0)	8 (57.1)	7 (77.8)	6 (85.7)
4	4 (13.3)	3 (21.4)	0	1 (14.3)

ECOG PS, Eastern Cooperative Oncology Group Performance Status.

a more than one symptom could be reported per patient

Fourteen patients entered the first cohort (total dose: 20 Gy), whilst nine patients were enrolled in the second cohort reaching a total dose of 40 Gy. Thereafter, seven patients were treated with three RT courses up to 60 Gy total dose. Among sixteen patients treated with more than one course, twelve (75%) needed a new contouring due to shrinkage tumour. Patients’ characteristics are shown in Table 1.

Regarding technique, nineteen patients (63.3%) were treated by photon beam 3D-CRT; in detail, twelve, three, and four patients were treated with a total dose of 20 Gy, 40 Gy, and 60 Gy, respectively. Eleven patients (36.7%) received an IMRT treatment due to clinical reasons; in particular, two, six, and three patients were enrolled in the first, second and third cohort, respectively.

Overall, only one (3.3%) acute toxicity G4 was registered (Table 2): a skin ulceration occurred two weeks after the treatment in a patient of the first cohort (total dose: 20 Gy) with a periorbital squamous cell cancer. Skin toxicity fully recovered two months later without topic therapy.

**Table 2.** Acute toxicity (RTOG)

	Overall *N* = 30	Cohort 1 *N* = 14	Cohort 2 *N* = 9	Cohort 3 *N* = 7
Skin				
G0 N (%)	13 (43.3)	8 (57.1)	4 (44.4)	1 (14.3)
G1 N (%)	13 (43.3)	4 (28.7)	3 (33.3)	6 (85.7)
G2 N (%)	3 (10.1)	1 (7.1)	2 (22.3)	0
≥G3 N (%)	1 (3.3)	1 (7.1)	0	0
Any grade N (%)	17 (56.6)	6 (42.8)	5 (55.6)	6 (85.7)
Oral Mucosa				
G0 N (%)	27 (90.0)	12 (85.8)	9 (100.0)	6 (85.7)
G1 N (%)	3 (10.0)	2 (14.2)	0	1 (14.3)
G2 N (%)	0	0	0	0
≥G3 N (%)	0	0	0	0
Any grade N (%)	3 (10.0)	2 (14.2)	0	1 (14.3)
Eye				
G0 N (%)	26 (86.7)	12 (85.8)	7 (77.7)	7 (100.0)
G1 N (%)	3 (10.0)	1 (7.1)	2 (22.3)	0
G2 N (%)	1 (3.3)	1 (7.1)	0	0
≥G3 N (%)	0	0	0	0
Any grade N (%)	4 (13.3)	2 (14.2)	2 (22.3)	0

No other G3 or worse toxicities were registered in overall population. Most patients (53.3%) experienced mild or moderate (G1-G2) skin toxicities: erythema, dry desquamation, and moderate oedema. Among three patients with G1 mucosal toxicity, two were treated in the mandibular region with a total dose of 20 Gy, whilst the other was enrolled in the third cohort for a squamous cell cancer in the cheek region. One patient enrolled in the first cohort reported a conjunctivitis graded as G2 requiring steroids after the irradiation of a periorbital lesion.

As far as late toxicity was concerned, only two patients (6.7%) reported a skin atrophy of the irradiated area graded as G1: both were treated with two courses of RT (total dose 40 Gy) six months earlier (data not shown).

**Figure 1.**
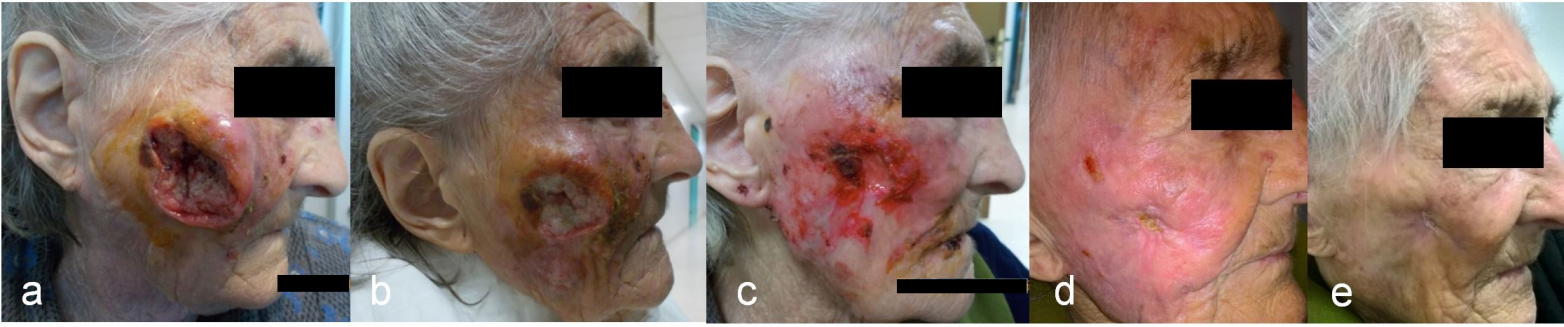
Skin cancer response over the treatment course. (**a**) Large ulcerated squamous cell carcinoma of the cheek; (**b**) the ulcer improved significantly after 20 Gy; (**c**) 40 Gy; (**d**) and 60 Gy; (**e**) 6 months after radiotherapy the lesion completely disappeared leaving a fibrotic scar.

Median follow-up time was six months. Overall symptoms-response rate was 90% including ten complete responses and seventeen partial responses. Two patients did not report any change in pain intensity, whilst a patient reported a worsening of pain in the irradiated lesion after treatment with a total dose of 20 Gy. In Cohort 1, the symptoms-response rate was 85.7%, whilst in cohort two it reached the 88.9%. In Cohort 3, we registered a symptoms response rate equal to 100%. Details about symptoms response rates are reported in Table 3. An example of disease response throughout the entire treatment is shown in Figure 1.

**Table 3.** Response rates

	Cohort 1 *N* = **14**	Cohort 2 *N* = **9**	Cohort 3 *N* = **7**
**CR N (%)**	4 (28.6)	2 (22.2)	4 (57.1)
**PR N (%)**	8 (57.2)	6 (66.7)	3 (42.9)
**NC N (%)**	1 (7.1)	1 (11.1)	0
**PD N (%)**	1 (7.1)	0	0
**ORR N (%)**	12 (85.8)	8 (88.9)	7 (100)

CR, Complete Response; NC, No change; ORR, Overall Response Rate; PD, Progression Disease; PR, Partial Response.

The mean pre- and post-treatment VAS was 2.8 and 2.1, respectively (*p* = 0.59). Fifteen out of the twenty patients symptomatic for pain before treatment reported partial or complete resolution, achieving 75% overall pain response rate. Furthermore, among these twenty patients, only three were on analgesic therapy at the time of radiation treatment and one of them discontinued it after RT.

The overall six-month symptom-free survival was 78.7% and 77.8% (*p* = 0.382) in patients treated with one course or more courses, respectively.

In our analysis, the PS, evaluated by ECOG scale, was improved or stable in 24 patients (80%). Moreover, the QoL, in terms of well-being (CLAS1), fatigue (CLAS2), and ability to perform daily activities (CLAS3) was improved or stable in 66.7% of patients.

## Discussion

In the present study, we report the safety and the feasibility of a repeated hypofractionated accelerated RT for NMSC in a particular setting represented by the older patients. One severe acute toxicity (3.3%) and two (6.7%) mild late toxicities were registered, so the DLT was not reached, not even when a total dose of 60 Gy in three cycles of four Gy per fraction, twice daily, was delivered.

This low toxicity profile is in line with the results of other experiences published in literature. Recently, a systematic review^10^ reported the state-of-the-art on palliative RT in NMSC, exploring six trials from 1984 to 2015.^9,29–33^ All cited studies showed a great tolerability of different schedules with an overall acute and late toxicities rates < 10%. Only one of these studies^9^ declared the pure palliative intent of the treatment which consisted of eight Gy per fraction delivered on days 0, 7, and 21 (0-7-21). This schedule was tested on an elderly patients population (median age: 91 years, range: 80–101 years) with poor performance status and difficulties getting to the radiotherapy centre. No data about the acute toxicity was available and no severe toxicity was observed.

The other studies^29–33^ reported few, if any, acute severe skin toxicities, even if total doses delivered were higher than ones usually used for palliative aim. As explained in a small case series with the review of the literature,^12^ in older patients with even moderately advanced NMSC a shorter or hypofractionated course of RT does not disadvantage them regarding outcome and should be considered an efficacious and tolerable treatment option. Various RT schemes have been tested, with generally good outcomes and few toxicities.^33–39^ The most often used regimen^33–36,38,39^ was weekly or bi-weekly irradiation with five to seven Gy per fraction for five to seven weeks. If we examine the biological equivalent dose (BED) used in the latter studies, we can note that it is similar to our BED (BEDα/β10: 83.3 Gy when seven Gy were repeated weekly for seven weeks versus BEDα/β10: 90.0 Gy, when three RT cycles were delivered). Indeed, these total doses are used to achieve local control and could be considered more radical in intent. An extreme example of ultra-hypofractionation is reported by Chan et al,^37^ where encouraging disease control was obtained by a single fraction RT in a younger population (median age: 68 years). The high delivered doses ranged from eighteen to 22.5 Gy and the crude ten-year late skin necrosis rate was 6%. Most of the skin necrosis healed spontaneously, with 16.7% of cases requiring surgical repair. The Authors themselves concluded that these doses are adequate for treatment of small superficial tumours, instead larger or deeper lesions could benefit from fractionated RT.

It could be argued that a total dose of 96 Gy in EQD2 (α/β3) could have a negative impact on late toxicities, such as cosmetic effect. It is difficult to draw firm conclusions about this aspect in our series, considering the relatively short median follow-up time, anyway both the old age and comorbidities could lessen this issue.

Regarding secondary outcomes, we registered an increasing symptoms response rate by increasing the number of cycles administered: the overall symptomatic response was 85.7% and 100% in patients treated once and in those receiving three cycles, respectively. These data suggest that in symptomatic patients, who are otherwise well, an immediate palliation could be associated with a better response, especially if the treatment is repeated more than once.

After the treatment, the QoL was stable or increased in two out of three patients, confirming the high reliability of such an approach in the older patient population.

The study presents some strengths and weaknesses: one could assume that the use of photons instead of electrons is questionable; indeed, the choice was driven by anatomical challenging sites and irregular anatomy that were treated. In these cases, dosimetry of photon beam is more reliable than electrons, as previously stated and documented.^40,41^

In addition to electron beam technique, brachytherapy and orthovoltage X-rays seem to be an option equally effective and safe in managing skin cancer,^42^ but it is already known that they are not so widespread as compared with linac-based radiotherapy.

Moreover, it may sound surprising that the PTV margins for 3D-CRT and IMRT were the same in this study, but this was done to guarantee homogeneity of treatment and patient comparison, as well as to be consistent with the literature. When 3D-conformal RT failed to satisfy constraints, the IMRT technique was used. However, it should be emphasized that the use of IMRT allows to reduce these margins increasing the feasibility of these treatments also in situations where tighter margins are necessary for the site of the lesions.

Finally, in our study, an oncogeriatric approach in the clinical management is lacking, however optimized clinical tools for the screening and comprehensive assessment of the older patient with NMSC, that could allow an effective distinction of “fit” from “frail” patients, are not yet available.^42,43^

In brief, this study demonstrated the feasibility of a split course RT reaching a total dose of 60 Gy, but, in our opinion, this schedule could be “adapted” as appropriate: in case of a symptomatic tumour needing a rapid palliation, even a single cycle of 20 Gy could be effective, but if a better response is pursued and if previous treatment has been well tolerated, the physician might decide to repeat the treatment. For this purpose, the timing is the key, because the split course allows the physician to evaluate the clinic course and decide accordingly.

Furthermore, it is important to note that this treatment could be “adaptive” not only to the patient’s clinical status, but even to the disease response: in our series a new CT-simulation and a new contouring was needed in 75% patient due to tumour shrinkage. Therefore, if more RT courses are planned, we recommend a close monitoring of disease in order to potentially reducing treatment fields and consequently side-effects.

In conclusion, in older patient population with NMSC unfit for surgery or chemoradiation, we reported the safety of a short accelerated hypofractionation, even when repeated. This approach could be considered as a treatment option if symptoms control is urgently required, and a long-lasting symptoms-free survival is pursued.
